# Applications of minimally invasive cardiac output monitors

**DOI:** 10.1186/1865-1380-5-18

**Published:** 2012-04-24

**Authors:** Jahan Porhomayon, Gino Zadeii, Samuel Congello, Nader D Nader

**Affiliations:** 1 VA Western New York Healthcare System, Division of Critical Care and Pain Medicine, Department of Anesthesiology, State University of New York at Buffalo School of Medicine and Biomedical Sciences, Buffalo, NY, USA; 2 University of Iowa, Mason City Cardiology, Mason City, Iowa, USA; 3 VA Medical Center, Rm 203C, 3495 Bailey Ave, Buffalo, NY 14215, USA

## Abstract

Because of the increasing age of the population, critical care and emergency medicine physicians have seen an increased number of critically ill patients over the last decade. Moreover, the trend of hospital closures in the United States t imposes a burden of increased efficiency. Hence, the identification of devices that facilitate accurate but rapid assessments of hemodynamic parameters without the added burden of invasiveness becomes tantamount. The purpose of this review is to understand the applications and limitations of these new technologies.

## Review

The ultimate goal of any hemodynamic monitoring system is to provide the clinicians with additional information on the underlying pathological condition and to guide fluid or vasopressor therapy. Cardiac output measurement and its response to therapeutic interventions are frequently used in critically ill patients. As the use of CO monitoring devices increases today, it is necessary to understand the application of such devices in different clinical settings. For many years pulmonary artery catheter (PAC) thermodilution cardiac output assessment was the monitor of choice for the management of critically ill patients. Thermodilution is a modification of the original indicator dilution techniques in which the injectate has a defined volume and temperature from which the thermodilution curve is generated [1]. As with the other indicator dilution techniques, CO is calculated from the area under the indicator thermodilution curve using the modified Stewart-Hamilton equation [2]. PAC was first used in dogs, and subsequently in humans 50 years later [2]. PAC provides valuable measurements, including right atrial pressure, right ventricular pressures, pulmonary artery pressures, pulmonary artery occlusive pressure, mixed venous saturation (SvO_2_), and CO. The derived hemodynamic variables are systemic and pulmonary vascular resistances. The major obstacle for the use of PAC has been the lack of demonstrating patient benefit and its level of invasiveness. Several prospective trials have demonstrated the lack of benefit from PACs. The PAC-man trial indicated that the routine placement of PACs had no effect on morbidity or mortality, and the ESCAPE trial found no difference in mortality or length of hospital stay when PAC parameters were compared with clinical assessment in the management of severe congestive heart failure patients [3-6]. Furthermore, for using PAC now, many physicians have lost the training, confidence, and familiarity with its use. PAC should probably be used only in selected patients by experienced practitioners. Contraindications to the insertion of PAC include tricuspid or pulmonary valve endocarditis/mechanical valve and right heart mass or thrombus. Like the PAC, each of these newer monitoring modalities requires education and training for effective use. For emergency room physicians, each technology provides a set of advantages and limitations. Minimally invasive cardiac output monitors allow for time efficiency in the emergency department setting and provide valuable information regarding the overall cardiovascular status of the patient. Declining cardiac index in trauma patients may indicate the need for revaluation of the patient. In general, early goal-directed therapy is usually better in the early phase of critical illness in contrast to late stages for sick patients. Minimally invasive monitoring devices for optimal CO and global oxygen balance may be of particular interest for emergency medicine physicians in the perioperative setting, acute lung injury, hypothermia induction, or preload and fluid responsiveness assessment in the management of septic shock and acute respiratory distress syndrome. Currently the evidence and literature have not necessarily caught up with the trends in the US and Europe with these devices, and partts of the article represent the authors' experience.

### Minimally invasive CO monitors

CO monitors use different principles for measuring CO. They include Doppler technology, echocardiography, pulse contour analysis, transpulmonary thermodilution, bioimpedance, bioreactance and Fick's principle.

### Esophageal Doppler

**Figure 1:**
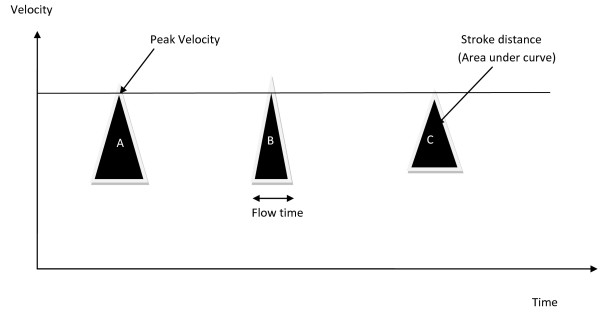
**Esophageal Doppler has the ability to measure flow time and peak velocity**. The flow time is the time from the beginning of aortic pulse waveform upstroke to its return to baseline. **A**. Peak velocity is a good indicator of myocardial contractibility (normal). **B**. The left ventricular ejection time (or flow-time) corrected for heart rate provides an index of preload (hypovolemia). **C**. Left ventricular failure. Note that during hypovolemia and heart failure the stroke distance is decreased.

Esophageal Doppler (ED) measuring aortic blood flow velocity was first introduced in 1971 [7]. The ED monitor measures the velocity of blood flow in the descending thoracic aorta using a flexible ultrasound probe. When combined with the aortic cross-sectional area it allows measurement of stroke volume and CO. The aortic diameter is obtained from a built-in nomogram or by direct measurement using M-mode echocardiography. A meta-analysis of several trials in critically ill patients showed high validity, but they were all performed in stable hemodynamic patients [8]. This meta-analysis suggested that ED was good at determining trends in CO but less effective in measuring absolute CO. The main application of this device has been for preload optimization, myocardial contractibility and goal-directed fluid therapy for surgical patients (Figure 1) [9-11]. ED can be safely utilized in emergency departments. In the study by Rodriguez et al. [12], they demonstrated in a prospective manner that cardiac output evaluation using ED in the emergency room was superior to physician estimation of cardiac output. He concluded that esophageal Doppler measurement of CO/CI appears to be practical from a physician time standpoint. Marquez et al. [13] demonstrated good correlation between ED and the LIDCO devices in cardiac surgery patients. Seoudi et al. prospectively investigated the correlation between PAC and ED in surgical trauma. On the basis of this study, it is reasonable to conclude that the ED is a valuable adjunct technology for CO and preload assessment for patients in the emergency medicine ward on mechanical ventilation, regardless of the level of mechanical ventilatory support [14]. The current literature supports the use of ED for assessment of cardiac output and left ventricular filling pressure [15-17]. This device is operator dependent, and placement of the probe in the wrong position can alter the CO reading. Additional limitations include the need for intubation of the trachea, expense, inaccurate CO in patients with aortic regurgitation, and the assumption that the division of the blood flow from the descending aorta is constant with even distribution to the brachiocephahlic and coronary arteries. This assumption is not always true in very ill patients.

### Echocardiography

**Figure 2:**
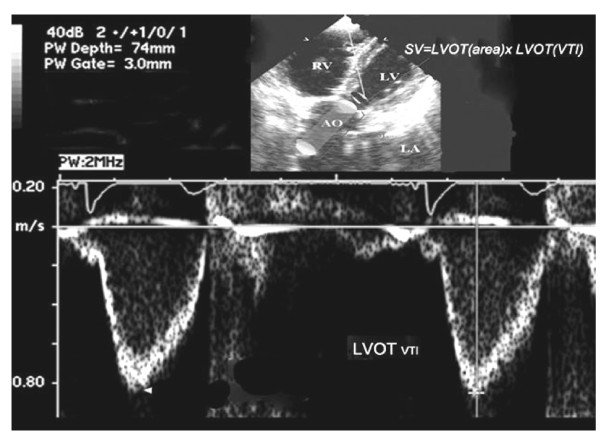
**Blood leaves the left ventricular outflow tract (LVOT) as a cylinder (aorta shown in *red*)**. The volume of the cylinder is equal to the stroke volume (*SV*). In order to calculate SV, the echocardiographer must obtain the diameter of the LVOT and velocity time integral (VTI) of the blood measured at the same exact location. *Ao *= Aorta, *LV *= left ventricle, *RV *= right ventricle, *LA *= left atrium.

Echocardiography has been used in the ICU and emergency medicine for many years to diagnose the underlying cause of hemodynamic instability. Like ED, echocardiography uses Doppler technology, but it relies on direct visualization of the cardiac anatomy and flow dynamics. The American College of Emergency Physicians encourages emergency medicine physicians to be able to rapidly diagnose pericardial tamponade and electromechanical dissociation, which represent truly emergent and potentially lethal cardiovascular conditions. A focused point of care exam will enable the emergency medicine physician to quickly assess: hemodynamic states [18] and unexplained hypotension [19], congestive heart failure, pericardial effusion, and pulmonary emboli [20]. In addition, it provides an emergency medicine physician an important tool to assess the efficiency of resuscitation and ultimately improve patient outcome [21]. Moreover, if the causes of circulatory failure are obscure, echocardiography provides the ability to evaluate structural abnormalities such as:

1. Wall motion abnormality for the diagnosis of myocardial injury [22] and evaluation of cardiac preload by estimating inferior vena cava (IVC) collapsibility [23].

2. Ventricular systolic dysfunction [24] and cardiac output (Figure 2).

One potential disadvantage of this device is the additional training required to make a proper diagnosis. Detailed cardiac echocardiography for diagnosis of complex cardiac diseases requires more advance training. Echocardiography measures CO only in a single point in time and is not suitable for trend analysis.

### Pulse contour analysis

Pulse contour analysis of cardiac output is based on the principles that stroke volume can be continuously estimated by analyzing the arterial pressure waveform obtained from an arterial line. The origin of the pulse contour method for estimation of the beat-to-beat stroke volume is based on the Windkessel model described by Otto Frank in 1899. In 1974, Wesseling et al. developed an algorithm that can be used to monitor stroke volume (SV) [25]. CO is calculated from the area under the curve of the systolic portion of the arterial pressure waveform divided by the aortic impedance multiplied by the heart rate. Currently, there are different commercially available devices that measure CO based on the pulse contour analysis method.

The most frequently used ones are the calibrated PiCCO monitor system (PULSION Medical Systems, Munich, Germany), the LiDCO monitoring system (LiDCO Ltd., London, UK), which is available as either a calibrated (LiDCOplus) or uncalibrated device (LiDCOrapid), and the uncalibrated FloTrac/Vigileo device (Edwards Life Sciences, Irvine, CA). The pulse contour devices are utilized with greater frequency in our local practice; hence, they are covered in more detail.

### PiCCO system

**Figure 3:**
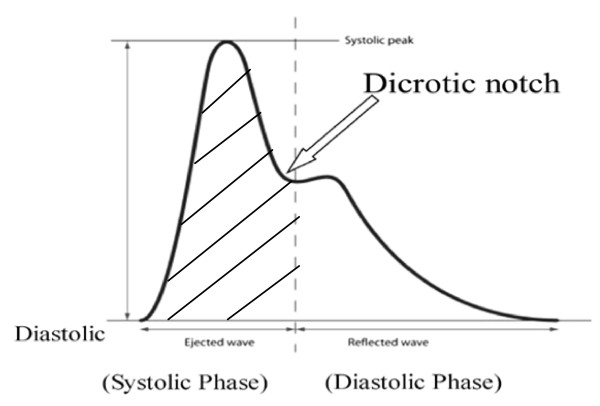
**PiCCO system: Stroke volume is the area under systolic portion of arterial pulse waveform (shaded)**. Stroke volume is calculated = [area under systolic phase (shaded) + aortic compliance] × shape of pressure curve.

The PiCCO system uses the pulse contour method based on the Wesseling algorithm for the calculation of CO. The system is periodically calibrated via the thermodilution method to calibrate the pulse pressure algorithm. PiCCO is a cardiac monitor that measures cardiac output and several volumes such as intrathoracic blood volume (ITBV), global end diastolic volume (GEDV), and extra vascular lung water (EVLW) [26]. PiCCO can also provide pulse contour parameters, which consist of continuous CO, systemic vascular resistance (SVR), stroke volume variation (SVV), and pulse pressure variation. The PiCCO system requires a thermistor-tipped central venous catheter and an arterial line usually introduced via the femoral, axillary, or brachial artery. After central venous injection of the cold indicator, the thermistor in the tip of the arterial catheter measures the downstream temperature changes. The CO is then calculated by analysis of the thermodilution curve using a modified Stewart-Hamilton algorithm. Pulse contour analysis continuously measures stroke volume and arterial pressure. CO and systemic vascular resistance (SVR) are calculated (Figure 3). Different studies in a variety of clinical settings have been performed in recent years validating the PiCCO system against intermittent pulmonary artery thermodilution (ITD) [27,28]. Goal-directed therapy with this technology has been reported in patients undergoing CABG surgery [29] and for preload optimization [30,31]. In the study by Uchino et al., the use of PiCCO was associated with a greater positive fluid balance and fewer ventilator-free days. After correction for confounding factors, the choice of monitoring did not influence major outcomes, whereas a positive fluid balance was a significant independent predictor of outcome [32]. This device has mainly been utilized in critical care setting but

PiCCO has the ability to measure (ITBV), (EVLW), and cardiac function index (CFI). These parameters are of interest as they are considered to be the most specific measures of cardiac preload, pulmonary edema, contractility, and a global indicator of cardiac performance. Therefore, PiCCO may give an emergency medicine physician a powerful tool for managing critically ill patients [29].

### Flo-Trac

Flo-Trac is another pulse contour CO monitoring system (Vigileo, Edwards Life Sciences) that was introduced in 2005. A special blood flow sensor, which is connected to an arterial line (radial, brachial, axillary or femoral artery), is needed. No external calibration is necessary [33-35]. This device calculates CO on a continuous basis by multiplying the pulse rate by calculated stroke volume. The direct relationship between arterial pulsatility and the stroke volume is used to calculate CO. Based on the model described by Langewouters et al. [36] individual demographics (body surface area, age, gender) are used for estimation of aortic compliance. Vascular compliance and resistance are determined using arterial waveform analysis. Several studies [33,37,38] have been performed concerning the accuracy of Vigileo CO monitoring in a variety of patients with different software versions of the device. The Vigileo monitor has been used in emergency medicine setting for resuscitation of burn victims with good result [39]. Stroke volume variation on this monitor can help clinicians to assess fluid responsiveness in the initial phase of septic shock. However, rapid changes in vascular motor tone may lead to impaired accuracy of CO monitoring. Flow-Trac accuracy is worse with arterial wave artifact, compromise of the arterial catheter, aortic regurgitation, intense peripheral vasoconstriction, irregular pulse, and severe cardiac hypo-function. Thus, its reliability is influenced by various conditions, especially in critically ill patients [40].

### Lithium dilution CO

The technique of using lithium dilution to measure CO was first described in 1993 by Linton et al. [41]. This technique uses pulse contour analysis for CO measurement and lithium dilution for system calibration. A small dose of lithium is injected into a peripheral vein, and an ion selective electrode is attached to a peripheral arterial line. The area under the curve of a plot of lithium concentration against time allows accurate calculation of the CO. The lithium dilution technique is of sufficient accuracy when there is constant blood flow and uniform mixing of blood. There are advantages and limitations to this technology, discussed in detail by Pearse et al. [42]. Other groups have demonstrated the accuracy of LIDCO [43]. Costa et al. showed good agreement among the LiDCO, PAC, and PICCO systems [44]. The major limitation to this device is the need for repetitive blood draws and, in the presence of neuromuscular blocking drugs, interference with calibration.

### Bioimpedance

The use of the electrical signal across the thorax to measure CO goes back to early 1970 [45]. Bioimpedance technology uses electrical resistance charges across the chest for identification of cyclic changes in blood flow. CO is then continuously estimated by analyzing beat-to-beat signal variation. CO calculation is based on different mathematical models. Early studies demonstrated only a fair correlation between thoracic electrical impedance (TEB) and thermodilution CO [46]. Despite many adjustments of the mathematical algorithms, validation studies continue to show mixed results [47]. In addition, TEB accuracy decreases with excessive lung water and pulmonary edema [48]. In recent years advances in bioimpedence technology have resulted in development of a novel technology placing the electrodes on endotracheal tube cuff (ECOM, CONMED, Utica, NY, USA). The proximity of the ascending aorta and trachea facilitated the design of this device. It can optimize the current delivery and signal recording from changes in the ascending aorta [49]. Because the tracheal mucosa produces mucous and fluid, the endotracheal cardiac output monitor (ECOM) electrodes have been designed to reduce the effects of fluid and mucous buildup. This device appears promising, and preliminary data indicate adequate reliability of this device [49-51]. The main disadvantage of ECOM is the need for an endotracheal tube and mechanical ventilation. ECOM can be used in emergency medicine for preload assessment and response to vasopressor therapy.

### Bioreactance

Bioreactance (NICOM; Cheetah Medical, Vancouver, WA, USA) is a unique technology for assessment of cardiac output [52]. It is similar to bioimpedance in that electrical current is applied to the chest via two leads. The bioreactance technique analyzes the frequency variations of the delivered oscillating current. This will result in a higher signal-to-noise ratio, and thus result in improved performance of the device. There have been several studies comparing the NICOM monitor to other monitors (PiCCO, Flo-Trac, PAC). These studies showed high agreement with the various monitors [52-56]. This device has very few known disadvantages and can be used in variety of settings.

### Fick's principle

Adolf Fick spent most of his life studying muscle metabolism, but in a brief publication in 1870, he described how mass balance might be used to measure cardiac output [57]. Later Guyton et al quoted the original work and expanded upon it [58]. It is based on the conservation of mass, such that the total uptake or release of a substance by an organ is the product of the blood flow to that organ multiplied by the arteriovenous concentration difference [59]. CO by modified Fick's method measures carbon dioxide (CO_2_) production and exhaled or end tidal CO_2 _at baseline and during a brief period of rebreathing. This will allow calculation of pulmonary artery blood flow. A new monitor called the NICO system (Novametrix Medical Systems, Wallingford, CT) uses Fick's equation for CO_2 _elimination. It is relatively noninvasive. Its principle states that over a fixed period of time the amount of CO_2 _leaving the lungs in the arterial blood is equal to the amount brought into the lungs in the venous blood minus the amount eliminated through the lungs. With this method, the CO is computed on breath-by-breath measurements of CO_2 _elimination. CO is proportional to the change in CO_2 _elimination divided by the change in end tidal CO_2 _resulting from a brief rebreathing period. Rebreathing measurements are made every 3 min for 35 s. The main drawback to this system is the assumption about the shunt fraction and arterial CO_2 _being equal to end-tidal CO_2_. Clinical and experimental data for CO determinations with the NICO monitor [60] give a better approximation of CO in patients who are less critically ill and have normal alveolar gas exchange. Additionally, preload optimization may be difficult using the NICO monitor, and caution should be exercised before using this monitor for fluid administration. Potentially large volumes of fluid may be administered to achieve desired endpoints [61]. Advantages of NICO include the easy setup and providing capillary blood flow and ventilator parameters, such as the ratio of tidal volume to dead space. The NICO monitor assumes that the partial venous CO_2 _concentration reflects the level of CO_2 _stored in the body. Therefore, any changes in metabolism or ventilation may alter the reliability of this monitor. Pulmonary shunting and heterogeneous ventilation decrease the precision of this device in acute lung injury since the shunt fraction is estimated from the concentration of the inspired fraction of oxygen and arterial oxygen saturation. Its main area of use has been in stable cardiac patients. This device can be utilized in the busy emergency medicine environment for evaluation of low cardiac output states if a patient is already intubated and on mechanical ventilation.

## Conclusions

**Figure 4:**
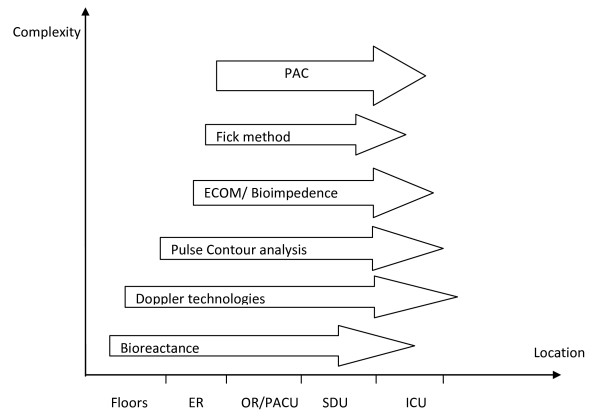
**Suggested use of cardiac output devices and monitoring system within the hospital system**. *ER *= Emergency room, *OR/PACU *= operating room/post-anesthesia care unit, *SDU *= step down unit, *ICU *= intensive care unit, *ECOM *= endotracheal cardiac output monitoring.

With an increasing need for utilization of hemodynamic monitoring due to the aging population, increased comorbidities and increasingly complex interventions and monitoring are becoming incorporated into the standard of care, and the need for hemodynamic monitoring is likely to increase. Because of the inherent limitations and complications of PAC in the busy emergency department, physicians are looking for less invasive devices to measure CO. There is no gold standard for the clinical measurement of CO. Therefore, comparison of these new technologies is somewhat challenging (Table 1). The level of invasiveness and complexity may help the clinicians decide where to best use the devices (Figure 4).

**Table 1:** Cardiac output monitors

	Advantages	Disadvantages
PAC		Pulmonary infarction
	Measure CVP	Rupture of pulmonary artery
	Intermittent and continuous	Arrhythmias
	SVR can be obtained	Need right heart catheterization

**Pulse wave** **Analysis**		

**A. PICCO**		
	Intermittent and continuous	Need a central venous access
	Measures GEDV/EVLW	
	Estimate preload	

**B. LIDCO**		
	Intermittent and continuous	Cannot be used if patient on lithium or NDM
	SVR can be obtained	Need frequent blood drawing
		Does not estimate preload

**C. Flo-trac**	SVR can be obtained	Not reliable in very high CO state
	Measure PPV/SVV	Perform poorly with tachyarrhythmia
	Many validation studies	Valvular pathology prevents accurate reading of CO

**Esophageal Doppler**	Less invasive	Needs intubated patient
	Simple to use	Only measure descending aortic flow
		Not good in AR

**Echocardiography**	Provides detailed cardiac information	Needs additional training
	Estimate preload	Inability to image patient

**Bioreactance**	Non-invasive	Numerous mathematical assumptions
	Continuous	Signal stability fails after 24 h
	Sensors can be placed anywhere in thorax and back	

**Bioimpedence**	Continuous	Numerous mathematical assumptions
	Difficult to set up	Signal stability fails after 24 h

**Flick's Principle**	Easy set up	Not suitable for unstable patient
	Provides additional ventilatory parameters	Shunt can affect CO estimation

*CVP *central venous pressure, *BP *blood pressure, *CO *cardiac output, *PCA *pulse contour analysis, *ICU *intensive care unit, *SVV *stroke volume variation, *PPV *pulse pressure variation, *GEDV *global end diastolic volume, *EVLW *extravascular lung water, *SVR *systemic vascular resistance, *CO *cardiac output, *AR *aortic regurgitation, *NDM *non-depolarizing muscle relaxant, *PAC *pulmonary artery catheter

## Competing interests

The authors do not have any financial and personal relationships with other people or organizations that could inappropriately influence (bias) their work. Examples of potential conflicts of interest include employment, consultancies, stock ownership, honoraria, paid expert testimony, patent applications/registrations, and grants or other funding.

## Authors' contributions

JP drafted the manuscript. GZ drafted the manuscript and made significant contributions to the revision. SC drafted the manuscript and made significant contributions to the revision. NN drafted the manuscript and provided figures. All authors read and approved the final manuscript.
